# Increased Ratio of Non-mercaptalbumin-1 Among Total Plasma Albumin Demonstrates Potential Protein Undernutrition in Adult Rats

**DOI:** 10.3389/fnut.2018.00064

**Published:** 2018-07-25

**Authors:** Yasuaki Wada, Yosuke Komatsu, Hirohisa Izumi, Takashi Shimizu, Yasuhiro Takeda, Masashi Kuwahata

**Affiliations:** ^1^Wellness & Nutrition Science Institute, Morinaga Milk Industry Co., Ltd., Zama, Japan; ^2^Department of Nutrition Science, Graduate School of Life and Environmental Sciences, Kyoto Prefectural University, Kyoto, Japan

**Keywords:** adult rat, dietary protein intake, low protein diet, mercaptalbumin, non-mercaptalbumin, plasma albumin, protein undernutrition, redox state of plasma albumin

## Abstract

The redox state of plasma albumin shifts in response to dietary protein intake in growing rats, and the shift is more sensitive than that of plasma albumin level, a classical marker of protein nutritional status. While it has been suggested that plasma albumin redox state could be useful as a novel marker of protein nutritional status, the above animal model is highly sensitive to dietary protein intake and the observation may not be extrapolated widely to humans. This study aimed to investigate whether albumin redox state also reflects protein nutritional status in adult rats, which have a lower dietary protein requirement and are less responsive to protein intake. Male adult rats were placed on AIN-93M diet (14% casein), or AIN-93M-based low protein diets (10 or 5% casein) *ad libitum* for 24 weeks. Whereas there was no significant difference in body weight between the groups at the end of the experimental period, the 5% casein diet group had the smallest gastrocnemius muscle weight among the groups, which was significantly lower than that of the 10% casein diet group. Plasma albumin level was also lower in the 5% casein diet group compared with the other groups, but the differences were limited and inconsistent during the experimental period. Among the albumin redox isoforms such as mercaptalbumin, non-mercaptalbumin-1, and non-mercaptalbumin-2, the ratio of non-mercaptalbumin-1 among total albumin was significantly higher in the 5% casein diet group, and the increase remained constant throughout the experimental period. Increased non-mercaptalbumin-1 ratio would thus demonstrate the presence of potential protein undernutrition in adult rats, as manifested only by a decreased gain in a specific type of skeletal muscle; non-mercaptalbumin-1 among total albumin ratio could be useful as a robust marker of protein nutritional status, contributing to prevention of protein undernutrition-related diseases such as frailty and sarcopenia.

## Introduction

Albumin represents the largest part of plasma proteins in mammals, comprising more than 50% of all plasma proteins (1). Human albumin constitutes of a single polypeptide chain of 585 amino acid residues with a molecular weight of approximately 66 kDa. It is exclusively synthesized in the liver and secreted into the circulation. Albumin synthesis is regulated by amino acid/protein nutritional status primarily at the transcriptional level, but it is also controlled post-transcriptionally, albeit to a lesser extent (2). Plasma albumin level has long been used as a marker of protein nutritional status; it is normally around 40 g/L, and a level of < 35 g/L is defined as hypoalbuminemia (3). Furthermore, regardless of implicated diseases, low plasma albumin level is considered to be a risk factors and a predictor of morbidity and mortality.

Human albumin has 35 cysteine (Cys) residues, of which 34 residues form 17 intramolecular disulfide bonds (2). The remaining Cys residue at position 34 (Cys34) remains free, which is conserved in all mammalian cases investigated to date. This residue is involved in the heterogeneity of albumin isoforms, i.e., mercaptalbumin (MA) with the reduced form of Cys34 and non-mercaptalbumin (NA) with the oxidized form of Cys34. NA is further categorized into two different states according to the modifications on Cys34, such as NA-1, having a mixed disulfide with low molecular weight thiols such as Cys, homo-Cys, or glutathione; and NA-2, having the thiol residue oxidized to sulfinic or sulfonic acid. These isoforms can be separated chromatographically (4, 5), and MA accounts for > 70% of the total plasma albumin in healthy adults (6). However, the ratio of MA among total albumin decreases with age (7, 8), liver diseases (9–12), renal failure (13–15), diabetes (16), and strenuous exercise (17–19), and these shifts in the albumin redox state have been attributed to oxidative stress accompanied by these diseases and physical situations. Recently, we reported that intake of a low protein diet shifted the redox state of plasma albumin to a more oxidized state in growing rats; the albumin redox state was shifted exclusively to NA-1 (20, 21). Plasma albumin redox state was more responsive to dietary protein intake compared with plasma albumin level (21). The shift of plasma albumin redox state, caused by protein insufficiency, was not related to oxidative stress. Furthermore, plasma albumin redox state correlated with albumin synthesis rate, which is stimulated by dietary amino acids/proteins (22–24).

Low protein intake is at times a prevalent public health problem, especially among older adults (25, 26), which can lead to frailty and sarcopenia (27, 28), and it is likely that novel biomarkers that sensitively demonstrate protein undernutrition would be of great help for prevention of these diseases. Plasma albumin redox state is apparently useful as a sensitive marker of protein nutritional status in humans, but the above observation was confirmed under limited conditions where growing rats were maintained under severe protein deficiency, i.e., a low protein diet containing 3% casein (CN) vs. a control diet containing 20% CN [AIN-93G, (29)]. This animal model has a high dietary protein requirement and is thus highly responsive to dietary protein nutritional intake (29), and the above-mentioned notion may only be extrapolated to limited situations of human nutrition.

Aiming to investigate whether the redox state of plasma albumin could be widely applied to humans as a marker for protein nutritional status, we tested the hypothesis that the redox state of plasma albumin would respond to dietary protein intake in adult rats, which have a lower dietary protein requirement and are less sensitive to protein nutritional status compared with growing rats (29). With reference to a detailed study on protein requirement in growing rats and taking into consideration lower protein requirement of adult rats (29, 30), adult Wistar rats were placed on AIN-93M diet (14% CN), or one of two AIN-93M-based low protein diets (10 or 5% CN) for 24 weeks. Plasma albumin and albumin redox state, as well as body and skeletal muscle weights, were compared between these dietary groups.

## Materials and methods

### Animal experiments

The study design was approved by the Animal Research Committee of Morinaga Milk Industry Co., Ltd. (protocol #16-042), and the experiments were performed in accordance with the committee's guideline. Eighteen male Wistar rats obtained commercially (14 weeks old; Japan SLC, Hamamatsu, Japan) were maintained individually in polycarbonate cages with wood shavings in a temperature-, humidity-, and light-controlled facility (21–25°C, 40–60% humidity, and a 12–h light/dark cycle), and allowed *ad libitum* access to water and AIN-93M diet (14% CN diet; Table 1), Oriental Yeast, Tokyo, Japan). After 1 week of acclimation to the facility conditions, (i.e., at 15 weeks old), animals were assigned to one of three different dietary groups (*n* = 6), and were fed *ad libitum* either a 14% CN diet, an AIN-93M-based iso-energetic diet containing 10% CN, or an AIN-93M-based diet containing 5% CN [designated as 10% CN diet and 5% CN diet, respectively (Table 1)]. These groups were maintained for 24 weeks. Approximately 50–200 μL of blood samples were collected from the lateral tail vein at weeks 0, 1, 2, 4, 8, 12, 16, 20, and 24, using syringes treated with ethylenediaminetetraacetic acid disodium (Sigma-Aldrich, St Louis, MO). Blood samples were centrifuged at 1700 × *g* for 10 min at room temperature, and the upper plasma layers were collected and stored at −80°C until analysis.

**Table 1:** Compositions of experimental diets.

	**14% CN diet (AIN-93M)**	**10% CN diet**	**5% CN diet**
**[INGREDIENT]**	**(g/kg diet)**
Casein	140	100	50
L-Cystine	1.80	1.28	0.64
Cornstarch	466	465	423
Dextrinized cornstarch	155	195	286
Sucrose	100	100	100
Cellulose	50	50	50
Soybean oil	40	40	40
*t*-Butylhydroquinone	0.008	0.008	0.008
AIN-93M mineral mixture	35	35	35
Mono-calcium phosphate ^a^	–	1.0	3.0
AIN-93 vitamin mixture	10	10	10
Choline bitartrate	2.5	2.5	2.5
**[NUTRIENTS]**	**(g/kg diet)**
Energy ^b^	14,790	14,790	14,786
Protein	127.4	91.4	46.4
Fat	43.5	43.4	43.0
Fiber	50.2	50.1	50.0

a *Decreased calcium content, accompanied by lower casein content, was compensated by adding mono-calcium phosphate*.

b *(kJ/kg diet)*.

After 24 weeks of the dietary treatments, animals were euthanized by deep anesthesia with sevoflurane. Blood was collected from the inferior vena cava, and plasma layers were collected after centrifugation. Liver samples were excised from the animals, weighed, and immediately frozen in liquid nitrogen. Soleus, plantaris, and gastrocnemius muscles were also excised and weighed. Plasma and liver samples were stored at −80°C until analysis.

### Plasma free amino acids

Free amino acid patterns were analyzed for plasma samples obtained from the inferior vena cava as described previously (21). Plasma samples were mixed with an equal volume of 10% trichloroacetic acid, and were centrifuged at 21500 × *g* for 15 min at 4°C to obtain soluble fractions. These fractions were filtered through 0.20-μm polyvinylidene fluoride filters (Thomson Instrument Company, Oceanside, CA), and filtrates were applied to an amino acid analyzer (L-8900; Hitachi High-Technologies, Tokyo, Japan) to determine the levels of all proteinogenic amino acids.

### Plasma albumin

Plasma samples, obtained during the experimental period from the lateral tail vein, were applied to a bromocresol green method (an A/G B test kit, Wako Pure Chemical, Osaka, Japan) to determine the albumin level.

### Redox state of plasma albumin

To examine the redox state of albumin, plasma samples obtained during the experimental period were subjected to HPLC analysis, as described previously (21). Briefly, MA, NA-1, and NA-2 were separated using a Shodex Asahipak ES-502N 7C column (Showa Denko, Kawasaki, Japan) maintained at 37°C. Albumin isoforms were eluted using a 100-min gradient with increasing ethanol concentrations from 0 to 10% in 0.4 M sodium sulfate and 50 mM sodium acetate (pH 4.85), with a flow rate of 0.5 mL/min. Fluorescence emission was measured at 340 nm for emission and 280 nm for excitation. Plasma samples were diluted to 1/120 with the above solvent (0% ethanol), and filtered through a 0.45 μm filter (Millipore, Billerica, MA). Sixty microliters of each filtrate was subjected to HPLC.

To calculate the ratio of MA, NA-1, and NA-2 among total albumin, the peak of the total albumin was vertically divided at the cross-points of their respective peaks. Peak integrations were allocated to MA, NA-1, and NA-2, from left to right, respectively.

### Hepatic albumin gene expression

Liver tissues were homogenized in TRIzol reagent (Life Technologies, Carlsbad, CA) using glass homogenizer tubes, and total RNA was extracted according to the instructions from the manufacturer. RNA samples were purified with an RNeasy mini kit (Qiagen, Hilden, Germany), and purity and quantity were confirmed using a NanoDrop ND-1000 (Nano-Drop Technologies, Wilmington, DE). Samples were then reverse-transcribed to cDNA using a High-Capacity cDNA Reverse Transcription Kit (Applied Biosystems, Foster City, CA).

Real-time PCR was performed with an ABI PRISM 7500 fast real-time PCR system (Applied Biosystems). Briefly, the reaction was performed using TaqMan universal master mix [No AmpErase UNG (2×)] with the PCR primer and probe set for the albumin gene (TaqMan Gene Expression Assays; Rn00592480_m1), and gene expression was normalized to an endogenous control gene, β-actin (Rn00667869_m1).

### Statistical analyses

All values are expressed as means ± SD (*n* = 6). Data were analyzed by one-way ANOVA followed by Tukey's post hoc test using JMP software (version 5.1.1; SAS Institute, Cary, NC). Significance was demonstrated at *P* < 0.05.

## Results

### Dietary energy/protein intake, body weight, and tissue weight

All the groups were fed their respective iso-energetic diets *ad libitum*. Energy and protein intakes are shown in Table 2. Energy intakes were significantly different between groups, and they were in the reverse order of protein contents in the diets. Protein intakes were in the order of protein contents in the diets, and were also significantly different between groups.

**Table 2:** Energy and protein intakes.

	**14% CN diet**	**10% CN diet**	**5% CN diet**
Energy (kJ/d)	230 ± 11^c^	251 ± 10^b^	296 ± 9^a^
Protein (g/d)	1.98 ± 0.09^a^	1.55 ± 0.06^b^	0.93 ± 0.03^c^

*Data are expressed as means ± SD (n = 6), which were analyzed by one-way ANOVA and Tukey's post hoc test. Values with different superscript letters are significantly different (P < 0.05). CN denotes casein*.

Body weights and tissue weights are shown in Table 3. Body weight increased continuously during the experimental period in all of the groups, and the 14% CN diet group had the lowest body weight among the three groups at week 24, but the differences did not reach statistical significance. Liver, soleus muscle, plantaris muscle, and gastrocnemius muscle were excised and weighed at the end of the experimental period. Only gastrocnemius muscle weight differed significantly between the groups, and the 5% CN diet group had the smallest weight among the three groups.

**Table 3:** Body and tissue weights.

	**14% CN diet**	**10% CN diet**	**5% CN diet**
**WEEK 0**			
Body Weight (g)	323.9 ± 5.1	324.3 ± 5.6	324.3 ± 9.4
**WEEK 24**			
Body Weight (g)	486.8 ± 20.2	513.0 ± 22.8	511.3 ± 8.5
**TISSUE WEIGHT (g/kg Bw)**			
Liver	29.8 ± 1.9	29.2 ± 1.9	28.8 ± 1.9
Soleus muscle	0.628 ± 0.043	0.620 ± 0050	0.639 ± 0.049
Plantaris muscle	1.650 ± 0.092	1.657 ± 0.107	1.560 ± 0.118
Gastrocnemius muscle	8.104 ± 0.331^ab^	8.224 ± 0.372^a^	7.633 ± 0.333^b^

*Data are expressed as means ± SD (n = 6), which were analyzed by one-way ANOVA and Tukey's post hoc test. Values with different superscript letters are significantly different (P < 0.05). BW and CN denote body weight and casein, respectively*.

### Plasma free amino acids

**Figure 1:**
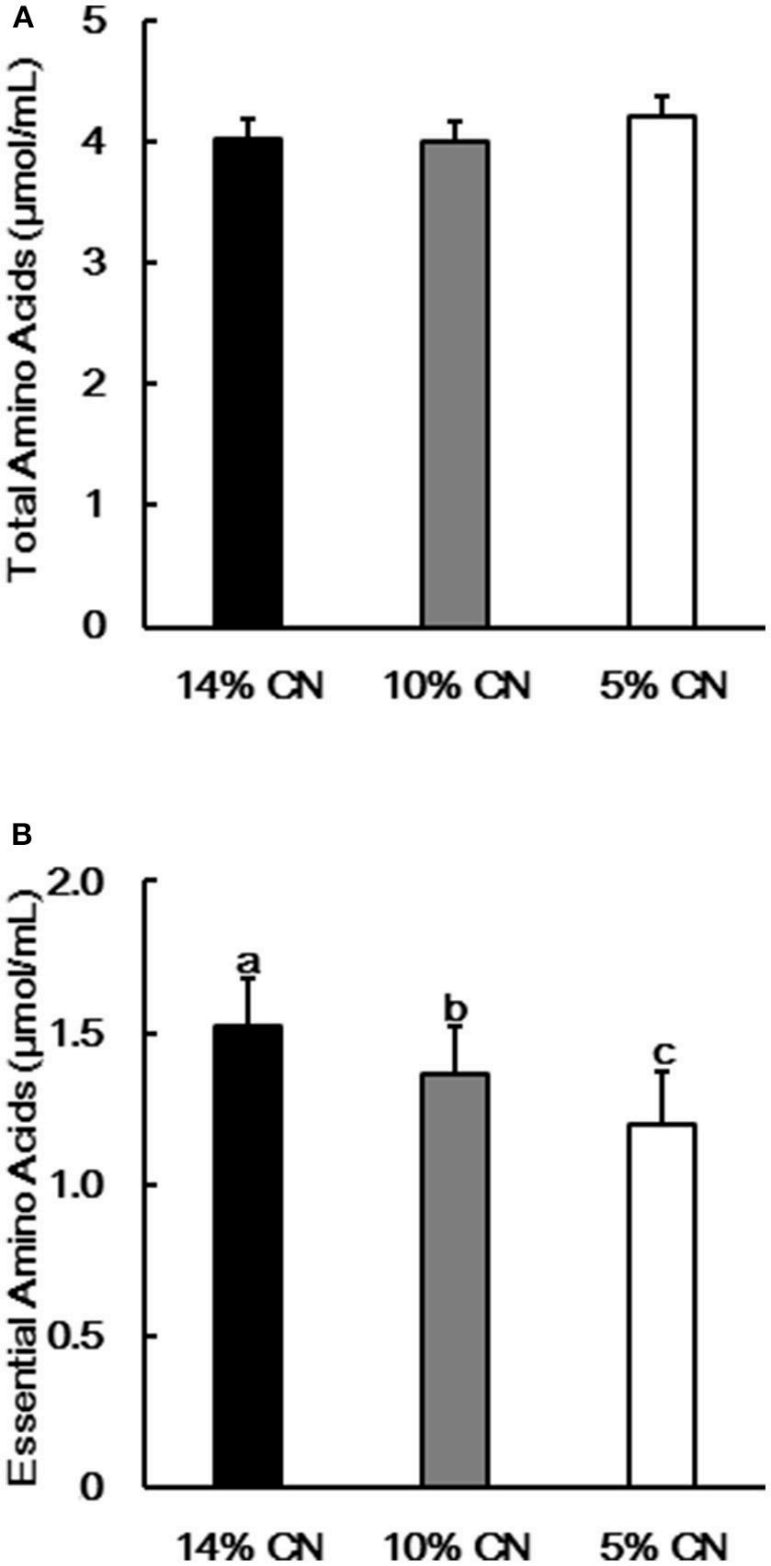
Plasma free total and essential amino acid levels. Concentrations of free total **(A)** and essential **(B)** amino acids were determined in plasma samples obtained at the end of the experimental period. Data are shown as means ± SD (*n* = 6). Values with different letters are significantly different (*P* < 0.05). CN denotes casein.

Free amino acid patterns were determined for plasma samples obtained at the end of the experimental period, as plasma free amino acids are influenced by immediate dietary protein and amino acid intakes (31). Whereas no significant difference in total amino acid levels was seen between the groups, levels of essential amino acids were significantly different (Figure 1); the levels were in the order of protein contents in the diets. A trend similar to that seen for total essential amino acids was observed for individual essential amino acids such as valine, leucine, isoleucine, methionine, phenylalanine, and tryptophan (Table 4).

**Table 4:** Plasma free amino acid levels.

**Amino acid**	**14% CN diet**	**10% CN diet (μmol/mL)**	**5% CN diet**
**ESSENTIAL**			
Val	0.253 ± 0.013^a^	0.214 ± 0.008^b^	0.174 ± 0.009^c^
Leu	0.179 ± 0.010^a^	0.159 ± 0.009^b^	0.143 ± 0.009^c^
Ile	0.111 ± 0.006^a^	0.098 ± 0.008^b^	0.081 ± 0.005^c^
Lys	0.399 ± 0.033^a^	0.334 ± 0.018^b^	0.335 ± 0.019^b^
Met	0.055 ± 0.008^a^	0.052 ± 0.003^a^	0.042 ± 0.002^b^
Phe	0.068 ± 0.004^a^	0.064 ± 0.003^a^	0.056 ± 0.003^b^
Trp	0.111 ± 0.006^a^	0.096 ± 0.007^b^	0.072 ± 0.007^c^
His	0.083 ± 0.008^a^	0.070 ± 0.001^b^	0.078 ± 0.004^a^
Thr	0.267 ± 0.032^a^	0.276 ± 0.023^a^	0.219 ± 0.019^b^
**NONESSENTIAL**			
Tyr	0.128 ± 0.011	0.117 ± 0.017	0.115 ± 0.012
Asp	0.008 ± 0.006	0.005 ± 0.005	0.012 ± 0.006
Asn	0.072 ± 0.006^a^	0.067 ± 0.003^ab^	0.062 ± 0.004^b^
Ser	0.234 ± 0.033^c^	0.303 ± 0.034^b^	0.535 ± 0.044^a^
Cys-Cys^1^	0.030 ± 0.013	0.035 ± 0.012	0.024 ± 0.011
Glu	0.163 ± 0.062	0.199 ± 0.091	0.158 ± 0.034
Gln	0.769 ± 0.042^a^	0.771 ± 0.049^a^	0.693 ± 0.044^b^
Pro	0.140 ± 0.025	0.129 ± 0.014	0.121 ± 0.019
Gly	0.169 ± 0.022^b^	0.189 ± 0.021^b^	0.308 ± 0.031^a^
Ala	0.686 ± 0.042^b^	0.725 ± 0.052^b^	0.881 ± 0.106^a^
Arg	0.105 ± 0.017	0.100 ± 0.013	0.097 ± 0.009

*Three-letter codes are used for all amino acids. Amino acid levels are expressed as means ± SD (n = 6), which were analyzed by one-way ANOVA and Tukey's post hoc test. Values with different superscript letters are significantly different (P < 0.05). CN denotes casein*. *^1^Cysteine levels were measured as cystine (Cys-Cys)*.

### Plasma albumin level and redox state of plasma albumin

**Figure 2:**
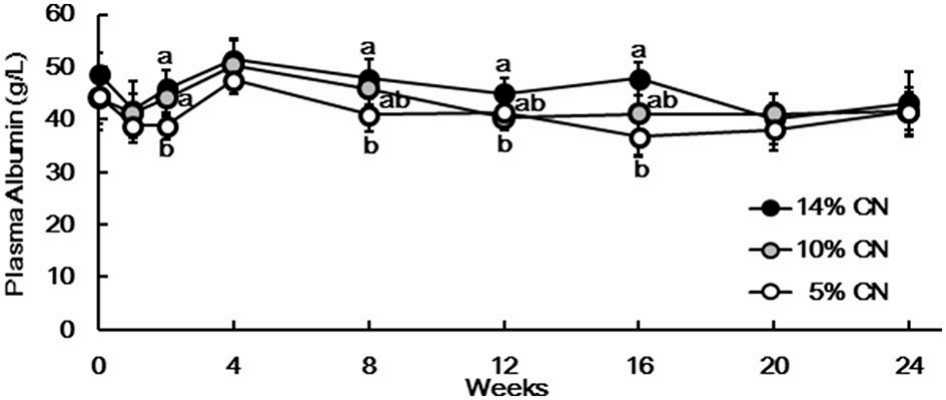
Plasma albumin levels. Albumin levels were measured for plasma samples obtained at weeks 0, 1, 2, 4, 8, 12, 16, 20, and 24. Data are expressed as means ± SD (*n* = 6). Values with different superscript letters at each time point are significantly different (*P* < 0.05). CN denotes casein.

Albumin levels were measured for plasma samples obtained during the experimental period. The levels differed between the groups only to limited extents throughout the experimental period (Figure 2). Albumin level of the 5% CN diet group tended to be lower than those of the other groups, but the differences were eventually dissolved later in the experimental period, such as at weeks 20 and 24.

**Figure 3:**
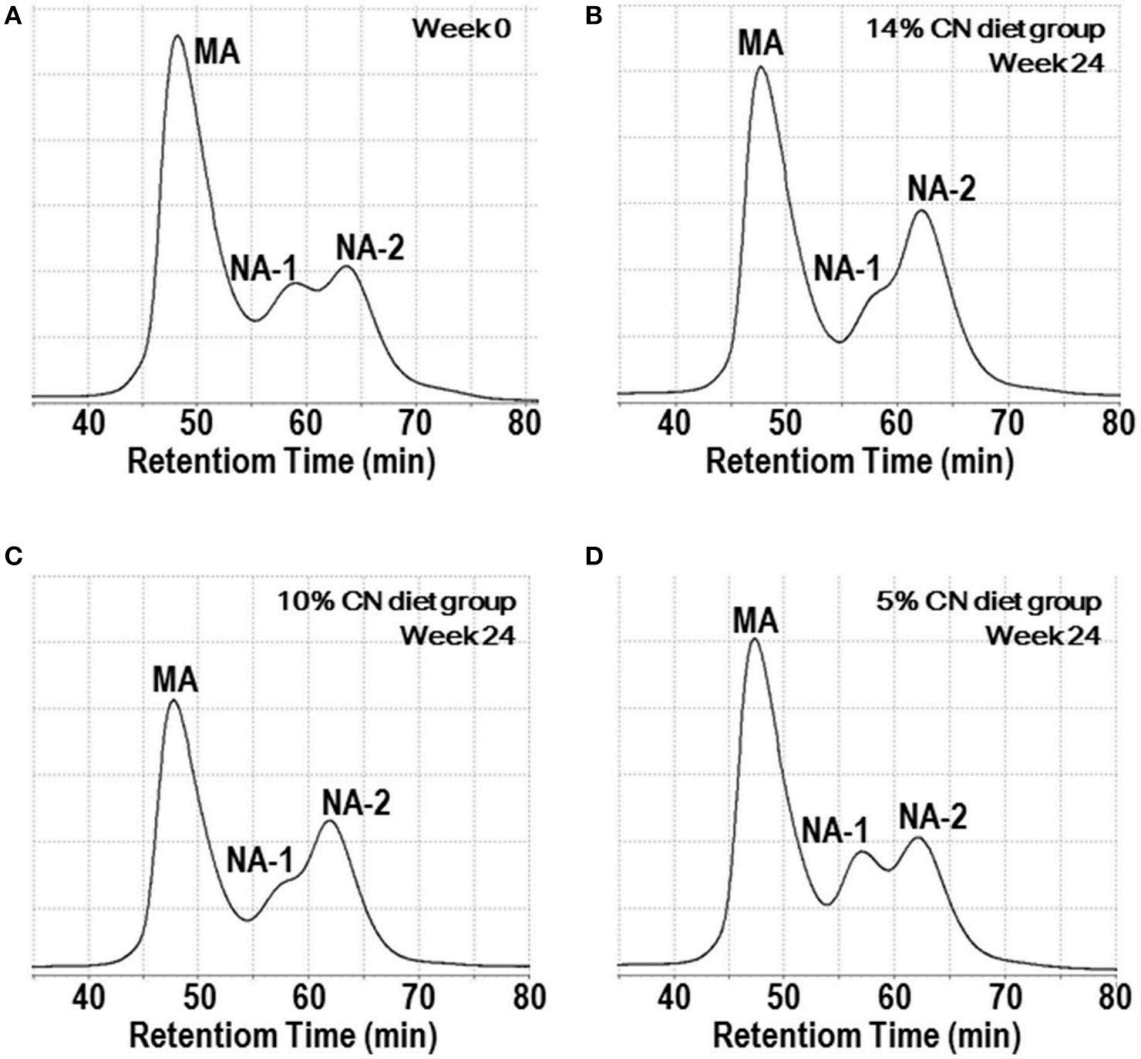
Chromatograms of plasma albumin redox state. Plasma samples, obtained at weeks 0, 1, 2, 4, 8, 12, 16, 20, and 24, were subjected to HPLC analysis to determine albumin redox state. Typical chromatograms at week 0 **(A)** and week 24 **(B–D)** are shown. CN, MA, and NA denote casein, mercaptalbumin, and non-mercaptalbumin, respectively.

**Figure 4:**
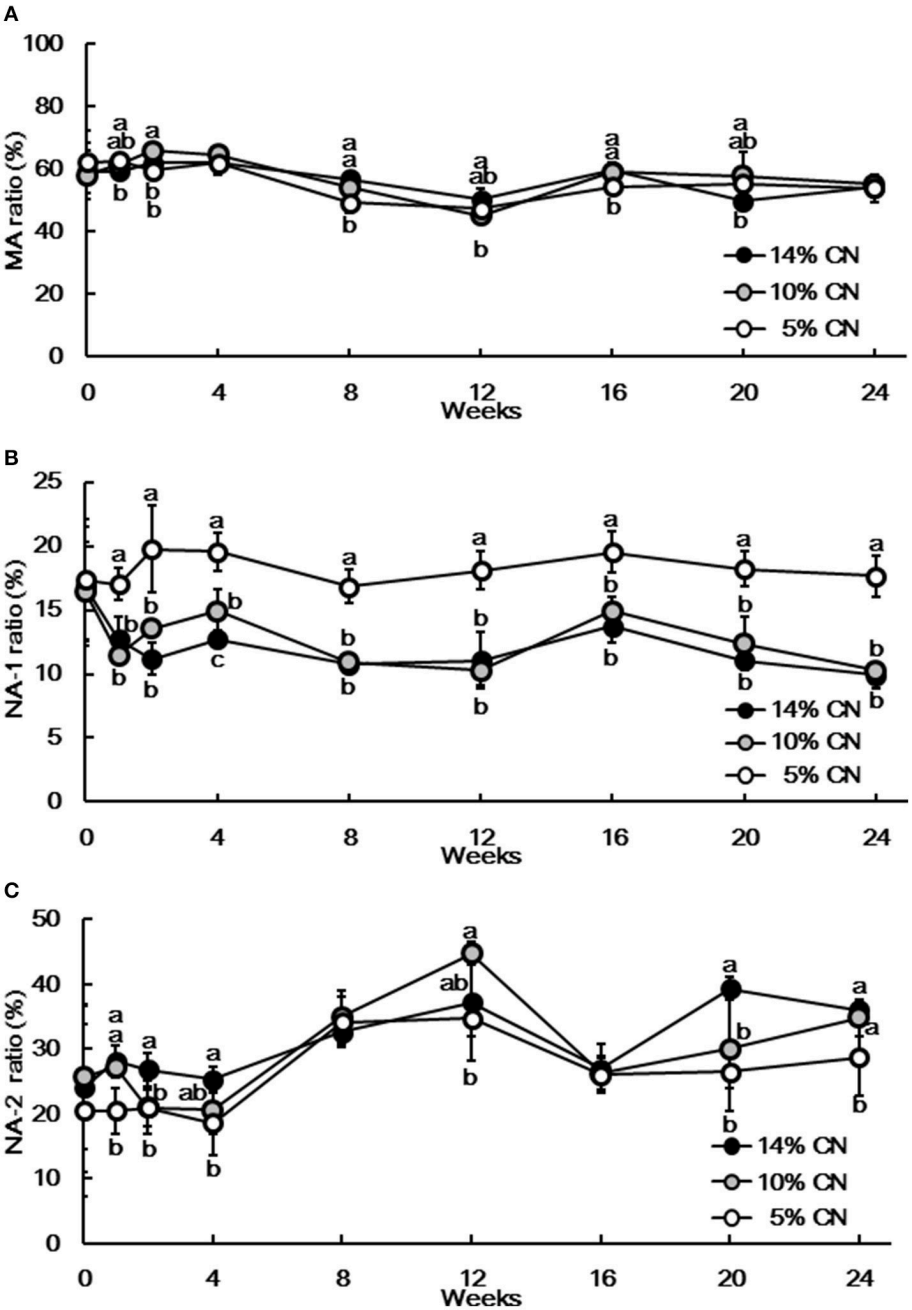
Ratios of mercaptalbumin, non-mercaptalbumin-1, and non-mercaptalbumin-2 among total plasma albumin. Plasma samples, obtained at weeks 0, 1, 2, 4, 8, 12, 16, 20, and 24, were subjected to HPLC analysis to determine albumin redox state. Ratios of mercaptalbumin **(A)**, non-mercaptalbumin-1 **(B)**, and non-mercaptalbumin-2 **(C)** among total albumin are expressed as means ± SD (*n* = 6). CN, MA, and NA denote casein, mercaptalbumin, and non-mercaptalbumin, respectively.

Plasma samples were also analyzed by HPLC to determine the albumin redox state (Figure 3). Albumin redox state was initially expressed as the ratio of MA among total albumin, as in our previous report (21). Similar to albumin levels, the differences in MA ratios were not evident throughout the experimental period (Figure 4A). Still, MA ratio of the 5% CN diet group appeared to be lower and the differences were significant at some of the time points. The redox state of plasma albumin was then scrutinized by expressing the ratio of NA-1 and NA-2 among total albumin, respectively. Notably, NA-1 ratios of the 5% CN diet group were significantly higher than those of the other two groups at all of the time points except at week 0 (Figure 4B). Furthermore, NA-2 ratios of the 5% CN diet group were the lowest at most of the time points (Figure 4C). Taken together, plasma albumin redox state of the 5% CN diet group was clearly different from those of the other diet groups; the redox state tended to exhibit a higher ratio of NA-1, accompanied by lower ratios of MA and NA-2.

### Hepatic albumin gene expression

**Figure 5:**
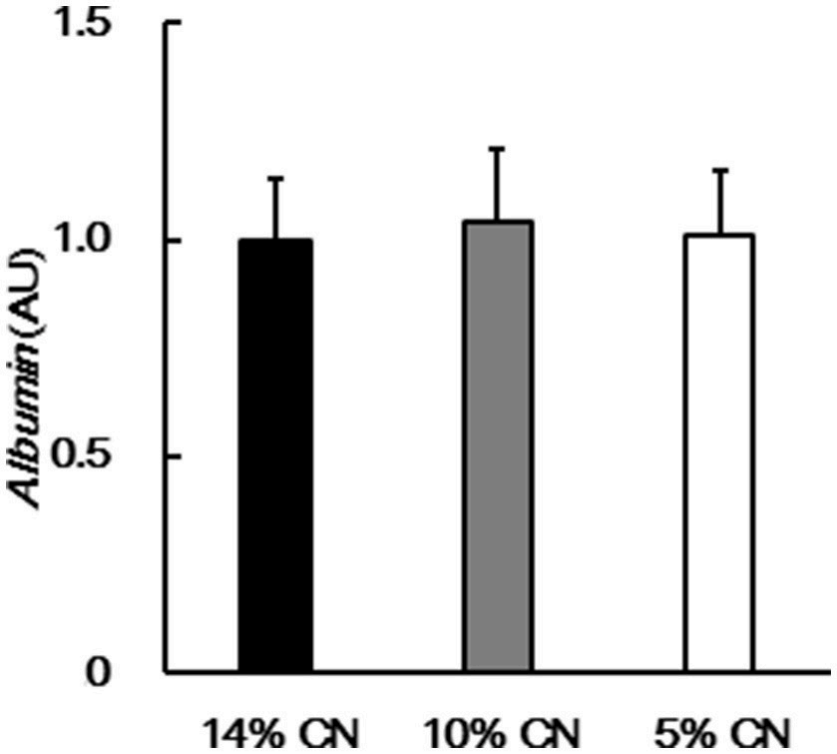
Hepatic albumin gene expression. Albumin gene expression was determined in livers obtained at the end of the experimental period, by performing real-time PCR. Data are shown as means ± SD (*n* = 6). No significant difference was seen between the groups (*P* < 0.05). CN denotes casein.

As albumin synthesis is primarily regulated at the transcription level (2), albumin gene expression was examined in the livers obtained at the end of the experimental period. No significant difference was seen between the groups (Figure 5).

## Discussion

We previously found that dietary protein intake modulated the redox state of plasma albumin via albumin turnover, including its synthesis rate, in growing rats (21). Here, we investigated whether this finding was applicable to adult rats, which are less responsive to dietary protein intake because of their lower dietary protein requirement (29), by placing them on AIN-93M-based diets with graded protein contents of 14, 10, and 5% CN.

Energy intakes were inversely associated with protein contents of the diets. This observation agrees with a previous study on growing rats fed graded levels of CN *ad libitum*, in which dietary intakes were observed to increase with moderate protein restriction (30). Still, protein intakes were proportional to protein contents of the diets and significantly different between the groups. When amino acid levels were measured at the end of the experimental period, essential amino acid levels differed significantly and they were in the order of protein contents in the diets. Decreased essential amino acid levels were also observed in our previous study on growing rats fed a low protein diet (21), and these differences in plasma free amino acid patterns are considered to reflect the differences in dietary protein intakes (31). Despite that, body weights of all of the groups increased during the experimental period and no significant difference was seen between them at the end of the experimental period. This apparently suggested that both the 10 and 5% CN diets would be sufficient for the maintenance of adult rats. However, when the weights soleus, plantaris, and gastrocnemius muscles were compared at the end of the experimental period as an assessment of protein nutritional status, gastrocnemius muscle weight of the 5% CN diet group was the lowest among the groups and was significantly smaller than that of the 10% CN diet group. This trend held true for plantaris muscle weight, but not soleus muscle weight, although the differences were not statistically significant. Gastrocnemius and plantaris muscles mainly consist of fast-twitch muscle fibers, whereas soleus muscle consists of a higher proportion of slow-twitch muscle fibers. When considering the fact that protein deficiency attenuates fast-twitch muscle fibers to greater extents than slow-twitch muscle fibers (32, 33), it was considered that the 5% CN diet was potentially insufficient for adult rats, which lead to insidious protein undernutrition, as manifested only by a decreased gain in a specific type of skeletal muscle mass after 24 weeks of the experimental period. Meanwhile, it is likely that the 10% CN diet, with its higher dietary intake, sustained the 24 weeks of maintenance in adult rats. Furthermore, although not statistically significant, gastrocnemius muscle weight of the 10% CN diet group was higher than that of the 14% CN diet group. This may suggest the higher efficiency of protein accretion in rats fed the 10% CN diet compared with the 14% CN diet, as indicated in a previous report showing that growing rats fed a 15% CN diet had higher protein efficiency than those fed a 20% CN diet (30). Histological study on gastrocnemius and plantaris muscles is likely more informative, requiring further investigation.

It was shown in our previous studies that both plasma albumin level and MA among total albumin ratio decreased significantly in growing rats fed a low protein diet (20, 21). Similarly, plasma albumin level and MA ratio were significantly lower in adult rats fed the 5% CN diet compared with those fed the 14 and 10% CN diets at some of the time points investigated. However, the differences in plasma albumin levels were quite limited, and were even dissolved later in the experimental period. MA ratios were fluctuant and did not exhibit any consistent trends during the experimental period. Thus, neither plasma albumin level nor MA ratio are likely to be useful to determine the presence of marginal protein undernutrition induced by the 5% CN diet. When plasma albumin redox state was assessed using NA-1 and NA-2 ratios, it was found that NA-1 ratio increased and NA-2 ratio decreased significantly in the 5% CN diet group, compared with the 14 and 10% CN diet groups. In Particular, higher NA-1 ratio was seen at as early as week 1 and the increase remained constant thereafter. It is notable that increased plasma NA-1 ratio and decreased NA-2 ratio were also seen in our previous study on growing rats maintained on a low protein diet (21). Taken together, it is likely that NA-1 is a robust marker that demonstrates the presence of insidious protein undernutrition. However, the NA-1 ratios calculated here should be treated with caution in terms of accuracy; the peaks of NA-1 and NA-2 overlapped to some extents, but our software does not have a function that can numerically separate them in order to calculate each NA-1 and NA-2 peak area; the calculation was made only by vertically dividing the overlapped peak at cross-points of the respective peaks.

It has been considered that albumin turnover, including albumin synthesis rate, could be responsible for the shift of albumin redox state in response to protein nutritional status (21). While albumin synthesis rate was not measured in this study, plasma free amino acid patterns (substances for albumin synthesis), and hepatic albumin gene expression levels would help understand albumin synthesis rate in adult rats. Levels of plasma free essential amino acids were significantly different between the groups, and the 5% CN diet group had the lowest level of essential amino acids, followed by the 10% CN diet group. On the other hand, although hepatic albumin gene expression is suppressed by protein deficiency (2), the gene expression levels were not significantly different between groups in our study. These observations are similar to those seen in our previous study (21); in energy-restricted growing rats, decreased free essential amino acid levels overwhelmed upregulated hepatic albumin gene expression, leading to a decrease in albumin synthesis rate. Thus, it can be considered that the 5% CN diet had a limited impact on protein nutritional status and hepatic albumin gene expression was not influenced, whereas ingestion of the 5% CN diet resulted in insufficiency of essential amino acids, which would slow down the albumin synthesis rate. Furthermore, if this is the case, the relationship between albumin synthesis rate and albumin redox state can be understood in the same way as was discussed previously (21); a marginal (but inconsistent) decrease in the MA ratio of the 5% CN diet group could be attributed to decreased *de novo* albumin synthesis; NA-2 isoform has a shorter half-life (34), whose clearance from the circulation would be facilitated by decreased *de novo* albumin synthesis; and NA-1 among total albumin ratio would be relatively increased as a consequence.

In conclusion, the ratio of plasma NA-1 among total albumin increased in response to moderate protein insufficiency in adult rats, and thus could serve as a robust marker that demonstrates the presence of potential protein undernutrition. Still, caution should be taken in extrapolating these findings to human nutrition, as plasma albumin turnover rates are markedly different between rats and humans (23, 35–37). Furthermore, effects of sex difference on the shift of albumin redox state have not been investigated to date. Despite these, there have been a couple of studies that suggest the shift of albumin redox state is mediated by changes of albumin synthesis rates; the shift of albumin redox state to a more oxidized state was partially dissolved by supplementation with branched-chain amino acids in liver disease patients, which supposedly occurred via improving albumin turnover (9, 12); and the shift of albumin redox state to a more oxidized state, caused by strenuous exercise-induced oxidative stress in athletes, was subsequently over-compensated for a day after the exercise training (18), suggesting an increase in albumin synthesis rate stimulated by exercise (38, 39). Further human trials are thus warranted to substantiate the relationship between the redox state of plasma albumin and protein nutritional status.

## Author contributions

YW, HI, TS, and YT designed the study. YW and YK performed experiments and analyzed data. YW and MK wrote the manuscript. YW had primary responsibility for the final content, and all authors read and approved the final manuscript.

### Conflict of interest statement

YW, YK, HI, TS, and YT are the employees of the study funders (Morinaga Milk Industry Co., Ltd.), but have no competing financial interests. The remaining author declares that the research was conducted in the absence of any commercial or financial relationships that could be construed as a potential conflict of interest.

## Abbreviations

CN: casein
Cys: cysteine
MA: mercaptalbumin
NA: non-mercaptalbumin.

## References

[1] Quinlan GJ Martin GS Evans TW. Albumin: biochemical properties and therapeutic potential. Hepatology (2005) 41:1211–9. 10.1002/hep.20720 15915465

[2] Wada Y Takeda Y Kuwahata M. Potential role of amino acid/protein nutrition and exercise in serum albumin redox state. Nutrients (2017) 10:E17. 10.3390/nu10010017 29295548 PMC5793245

[3] Gatta A Verardo A Bolognesi M. Hypoalbuminemia. Intern Emerg Med. (2012) 7(Suppl. 3):S193–9. 10.1007/s11739-012-0802-0 23073857

[4] Hayashi T Era S Kawai K Imai H Nakamura K Onda E Observation for redox state of human serum and aqueous humor albumin from patients with senile cataract. Pathophysiology (2000) 6:237–43. 10.1016/S0928-4680(99)00022-X

[5] Hayashi T Suda K Imai H Era S. Simple and sensitive high-performance liquid chromatographic method for the investigation of dynamic changes in the redox state of rat serum albumin. J Chromatogr B Analyt Technol Biomed Life Sci. (2002) 772:139–46. 10.1016/S1570-0232(02)00068-5 12016025

[6] Kubota K Nakayama A Takehana K Kawakami A Yamada N Suzuki E. A simple stabilization method of reduced albumin in blood and plasma for the reduced/oxidized albumin ratio measurement. Int J Biomed Sci. (2009) 5:293–301. 23675150 PMC3614789

[7] Era S Kuwata K Imai H Nakamura K Hayashi T Sogami M. Age-related change in redox state of human serum albumin. Biochim Biophys Acta. (1995) 1247:12–6. 10.1016/0167-4838(94)00166-E 7873580

[8] Oettl K Marsche G. Redox state of human serum albumin in terms of cysteine-34 in health and disease. Methods Enzymol. (2010) 474:181–95. 10.1016/S0076-6879(10)74011-8 20609911

[9] Fukushima H Miwa Y Shiraki M Gomi I Toda K Kuriyama S. Oral branched-chain amino acid supplementation improves the oxidized/reduced albumin ratio in patients with liver cirrhosis. Hepatol Res. (2007) 37:765–70. 10.1111/j.1872-034X.2007.00123.x 17573945

[10] Oettl K Stadlbauer V Petter F Greilberger J Putz-Bankuti C Hallström S. Oxidative damage of albumin in advanced liver disease. Biochim Biophys Acta. (2008) 1782:469–73. 10.1016/j.bbadis.2008.04.002 18498776

[11] Domenicali M Baldassarre M Giannone FA Naldi M Mastroroberto M Biselli M. Posttranscriptional changes of serum albumin: clinical and prognostic significance in hospitalized patients with cirrhosis. Hepatology (2014) 60:1851–60. 10.1002/hep.27322 25048618

[12] Setoyama H Tanaka M Nagumo K Naoe H Watanabe T Yoshimaru Y. Oral branched-chain amino acid granules improve structure and function of human serum albumin in cirrhotic patients. J Gastroenterol. (2017) 52:754–65. 10.1007/s00535-016-1281-2 27873095 PMC5437197

[13] Terawaki H Yoshimura K Hasegawa T Matsuyama Y Negawa T Yamada K. Oxidative stress is enhanced in correlation with renal dysfunction: examination with the redox state of albumin. Kidney Int. (2004) 66:1988–93. 10.1111/j.1523-1755.2004.00969.x 15496170

[14] Mera K Anraku M Kitamura K Nakajou K Maruyama T Otagiri M. The structure and function of oxidized albumin in hemodialysis patients: Its role in elevated oxidative stress via neutrophil burst. Biochem Biophys Res Commun. (2005) 334:1322–8. 10.1016/j.bbrc.2005.07.035 16054887

[15] Regazzoni L Del Vecchio L Altomare A Yeum KJ Cusi D Locatelli F. Human serum albumin cysteinylation is increased in end stage renal disease patients and reduced by hemodialysis: mass spectrometry studies. Free Radic Res. (2013) 47:172–80. 10.3109/10715762.2012.756139 23215783

[16] Oettl K Reibnegger G Schmut O. The redox state of human serum albumin in eye diseases with and without complications. Acta Ophthalmol. (2011) 89:e174–9. 10.1111/j.1755-3768.2009.01824.x 20064117

[17] Imai H Hayashi T Negawa T Nakamura K Tomida M Koda K. Strenuous exercise-induced change in redox state of human serum albumin during intensive kendo training. Jpn J Physiol. (2002) 52:135–40. 10.2170/jjphysiol.52.135 12139771

[18] Imai H Era S Hayashi T Negawa T Matsuyama Y Okihara K Effect of propolis supplementation on the redox state of human serum albumin during high-intensity kendo training. Adv Exerc Sports Physiol. (2005) 11:109–13.

[19] Lamprecht M Greilberger JF Schwaberger G Hofmann P Oettl K. Single bouts of exercise affect albumin redox state and carbonyl groups on plasma protein of trained men in a workload-dependent manner. J Appl Physiol. (2008) 104:1611–7. 10.1152/japplphysiol.01325.2007 18420715

[20] Kuwahata M Hasegawa M Kobayashi Y Wada Y Kido Y. An oxidized/reduced state of plasma albumin reflects malnutrition due to an insufficient diet in rats. J Clin Biochem Nutr. (2017) 60:70–5. 10.3164/jcbn.16-33 28163385 PMC5281528

[21] Wada Y Sato Y Miyazaki K Takeda Y Kuwahata M. The reduced/oxidized state of plasma albumin is modulated by dietary protein intake partly via albumin synthesis rate in rats. Nutr Res. (2017) 37:46–57. 10.1016/j.nutres.2016.12.003 28215314

[22] De Feo P Horber FF Haymond MW Meal stimulation of albumin synthesis: a significant contributor to whole body protein synthesis in humans. Am J Physiol. (1992) 263(4 Pt 1):E794–9. 10.1152/ajpendo.1992.263.4.E794 1415702

[23] Caso G Feiner J Mileva I Bryan LJ Kelly P Autio K. Response of albumin synthesis to oral nutrients in young and elderly subjects. Am J Clin Nutr. (2007) 85:446–51. 10.1093/ajcn/85.2.446 17284742

[24] Thalacker-Mercer AE Johnson CA Yarasheski KE Carnell NS Campbell WW Nutrient ingestion, protein intake, and sex, but not age, affect the albumin synthesis rate in humans. J Nutr. (2007) 137:1734–40. 10.1093/jn/137.7.1734 17585023 PMC3885871

[25] Mendonca N Granic A Mathers JC Hill TR Siervo M Adamson AJ. Prevalence and determinants of low protein intake in very old adults: insights from the Newcastle 85+ Study. Eur J Nutr. (2017). 10.1007/s00394-017-1537-5. [Epub ahead of print]. 28948346 PMC6267410

[26] Hamamatsu Y Goto C Nishitani M Shimadate R Ueno J Kusakari Y. Associations between neighborhood food environments and deficient protein intake among elderly people in a metropolitan suburb: A case study in Kisarazu city, Japan. Am J Hum Biol. (2017) 29:6. 10.1002/ajhb.23043 28719103

[27] Evans WJ Paolisso G Abbatecola AM Corsonello A Bustacchini S Strollo F. Frailty and muscle metabolism dysregulation in the elderly. Biogerontology (2010) 11:527–36. 10.1007/s10522-010-9297-0 20683658

[28] Kim JS Wilson JM Lee SR. Dietary implications on mechanisms of sarcopenia: roles of protein, amino acids and antioxidants. J Nutr Biochem. (2010) 21:1–13. 10.1016/j.jnutbio.2009.06.014 19800212

[29] Reeves PG Nielsen FH Fahey GC Jr. AIN-93 purified diets for laboratory rodents: final report of the American Institute of Nutrition ad hoc writing committee on the reformulation of the AIN-76A rodent diet. J Nutr. (1993) 123:1939–51. 10.1093/jn/123.11.1939 8229312

[30] Du F Higginbotham DA White BD. Food intake, energy balance and serum leptin concentrations in rats fed low-protein diets. J Nutr. (2000) 130:514–21. 10.1093/jn/130.3.514 10702578

[31] Young VR Marchini JS Cortiella J. Assessment of protein nutritional status. J Nutr. (1990) 120(Suppl. 11):1496–502. 10.1093/jn/120.suppl_11.1496 2243295

[32] Veldee MS Peth LD. Can protein-calorie malnutrition cause dysphagia? Dysphagia (1992) 7:86–101. 10.1007/BF02493439 1572231

[33] Kobayashi Y Somoto Y Mitsuyama E Tanaka A Yuda N Nakada H Supplementation of protein-free diet with whey protein hydrolysates prevents skeletal muscle mass loss in rats. J Nutr Intermediary Metab. (2016) 4:1–5. 10.1016/j.jnim.2016.03.001

[34] Iwao Y Anraku M Hiraike M Kawai K Nakajou K Kai T. The structural and pharmacokinetic properties of oxidized human serum albumin, advanced oxidation protein products (AOPP). Drug Metab Pharmacokinet. (2006) 21:140–6. 10.2133/dmpk.21.140 16702734

[35] Papet I Dardevet D Sornet C Béchereau F Prugnaud J Pouyet C. Acute phase protein levels and thymus, spleen and plasma protein synthesis rates differ in adult and old rats. J Nutr. (2003) 133:215–9. 10.1093/jn/133.1.215 12514293

[36] Jeffay H Winzler RJ. The metabolism of serum proteins. II. The effect of dietary protein on the turnover of rat serum protein. J Biol Chem. (1958) 231:111–6. 13538953

[37] Prinsen BH de Sain-van der Velden MG. Albumin turnover: experimental approach and its application in health and renal diseases. Clin Chim Acta. (2004) 347:1–14. 10.1016/j.cccn.2004.04.005 15313137

[38] Sheffield-Moore M Yeckel CW Volpi E Wolf SE Morio B Chinkes DL. Postexercise protein metabolism in older and younger men following moderate-intensity aerobic exercise. Am J Physiol Endocrinol Metab. (2004) 287:E513–22. 10.1152/ajpendo.00334.2003 15149953

[39] Sheffield-Moore M Paddon-Jones D Sanford AP Rosenblatt JI Matlock AG Cree MG. Mixed muscle and hepatic derived plasma protein metabolism is differentially regulated in older and younger men following resistance exercise. Am J Physiol Endocrinol Metab. (2005) 288:E922–9. 10.1152/ajpendo.00358.2004 15644460

